# The lost scent of excitation: dissociation between cognitive desire and physical arousal in Kallmann syndrome

**DOI:** 10.1210/jendso/bvag247

**Published:** 2026-10-22

**Authors:** Giuseppe Seminara, Ettore D’Aleo, Marco Leuzzi, Maria Carmela Zagari, Emanuela A Greco, Antonio Aversa

**Affiliations:** Department of Experimental and Clinical Medicine, Magna Graecia University of Catanzaro, Catanzaro 88100, Italy; Endocrinology Unit, Renato Dulbecco University Hospital, Catanzaro 88100, Italy; Dipartimento di Scienze Economiche, Psicologiche, della Comunicazione, della Formazione e Motorie, Niccolò Cusano University, Rome 00166, Italy; Department of Experimental and Clinical Medicine, Magna Graecia University of Catanzaro, Catanzaro 88100, Italy; Department of Experimental and Clinical Medicine, Magna Graecia University of Catanzaro, Catanzaro 88100, Italy; Dipartimento di Scienze Economiche, Psicologiche, della Comunicazione, della Formazione e Motorie, Niccolò Cusano University, Rome 00166, Italy; Department of Experimental and Clinical Medicine, Magna Graecia University of Catanzaro, Catanzaro 88100, Italy; Endocrinology Unit, Renato Dulbecco University Hospital, Catanzaro 88100, Italy

**Keywords:** Kallmann syndrome, olfaction, sexual desire, hormonal replacement therapy, psychosexual profile

## Abstract

Kallmann syndrome (KS) is a rare form of congenital hypogonadotropic hypogonadism (CHH) that causes delayed puberty and infertility, accompanied by a lifelong loss of smell. While hormone replacement therapy (HRT) restores standard hormone levels, the specific impact of an absent sense of smell on sexual relationships and desire remains largely unstudied. This study examined how lifelong smell loss shapes the psychosexual experiences of affected individuals. 38 adults were evaluated: 19 patients receiving stable HRT (10 with KS who lacked a sense of smell and 9 with CHH but normal smell) and 19 healthy matched individuals. Participants completed validated questionnaires assessing sexual desire, physical arousal, romantic behaviors, and attitudes toward intimacy. HRT effectively restored baseline sexual desire and general attitudes across all patients. However, individuals with KS reported significantly lower physical sexual arousal and engaged in far fewer sexual behaviors than those with an intact sense of smell. The lack of smell independently predicted lower arousal levels, despite patients maintaining normal mental desire and positive attitudes toward sex. These findings show that hormone therapy alone is insufficient to fully restore the sexual experience in KS. The lifelong absence of smell creates a distinct divide between mental desire and physical arousal, undermining sexual confidence and intimacy. Healthcare providers should therefore incorporate psychosexual counseling into routine clinical care to help patients navigate these sensory and emotional challenges.

Congenital hypogonadotropic hypogonadism (CHH) is a rare disorder (1:10 000 live births) characterized by a deficiency in the production, secretion, or action of gonadotropin-releasing hormone (GnRH), leading to delayed puberty and infertility (1). Clinically, CHH in males is characterized by absent or minimal virilization, poor libido, and erectile dysfunction, with cryptorchidism and microphallus being typical in infant boys (2). Adult females with CHH typically present with primary amenorrhea, and both sexes may exhibit eunuchoid proportions (3). When anosmia or hyposmia is present, CHH is diagnosed as Kallmann syndrome (KS), which results from a disruption in the embryonic migration of GnRH neurons from the olfactory placode to the hypothalamic nasal region, where these neurons normally settle and differentiate permanently (4). The prevalence of KS is estimated to be 1 in 40 000 in females and 1 in 8000 in males (5).

Clinical studies examining patients with smell disorders support the hypothesis that absence of olfaction may have a specific and negative long-term impact on core components of the sexual experience, such as the intensity of orgasm (6, 7). Among eugonadal individuals, hyposmic men have significantly lower International Index of Erectile Function scores, while hyposmic women score significantly lower across all domains of the female sexual function index (8). Beyond their metabolic and reproductive sequelae, chronic olfactory disorders have been repeatedly associated with reduced quality of life, higher depressive symptoms, impaired social functioning, and disruption of intimate and sexual relationships (9-13). Patients frequently report diminished sexual enjoyment and a heightened sense of relational insecurity, underscoring that anosmia may erode not only sexual functioning but also core aspects of identity, self-esteem, and partnership stability (14-16).

Treatment with exogenous gonadotropins induces puberty and fertility in CHH patients, while treatment with sex hormones restores eugonadism (17), but in those with KS olfaction cannot be restored, as the olfactory defect results from structural absence of olfactory neurons. The lifelong anosmia in these patients may impact sexual and social experience beyond the endocrine dysfunction, though this dimension has received no systematic study and it has not been disentangled from the global manifestations of the syndrome. Normosmic hypogonadotropic hypogonadism (nHH) may provide comparisons with KS, suggesting if anosmia could contribute independently to social and sexual difficulties beyond those attributable to hypogonadism alone.

In this context, the aim of the present cross-sectional study was to investigate the extent to which the olfactory component in patients with KS contributes to determining sexual desire and relationships, compared with patients with nHH and healthy controls.

## Materials and methods

### Study design and objective

The present observational cross-sectional study has been designed to distinguish the impact of endocrine dysfunction of CHH from the specific contribution of olfactory deficit of KS on sexual desire and social-sexual relationships. The study aimed to compare KS patients, patients with nHH, and healthy controls.

This cross-sectional observational study was conducted in accordance with the ethical principles outlined in the Declaration of Helsinki. The study followed the strengthening the reporting of observational studies in epidemiology (STROBE) guidelines for observational studies (18).

### Participants and eligibility criteria

Study participants belonging to KS and nHH groups were recruited from the rare endocrinological disease outpatient clinic at the “Renato Dulbecco” University Hospital of Catanzaro. The clinical cohort comprised adult patients (age > 18 years) with a confirmed diagnosis of CHH, including both KS and nHH phenotypes. Diagnosis of CHH was established according to the clinical, hormonal, and radiological criteria of the European Consensus Statement (19). According to the results of the Sniffin’ Sticks-Extended test (20, 21), patients with CHH were classified as nHH (normosmic) or KS (hypo/anosmic). Patients with CHH were categorized based on the most recent validated threshold-discrimination-identification (TDI) sum score cutoffs (22). Specifically, anosmia was defined as a TDI score ≤16.75, hyposmia was defined by scores ranging from 17 to 30, and normosmia was identified by a score ≥30 (22).

To ensure the homogeneity of the sample and minimize confounding variables related to hormonal fluctuations, strict inclusion criteria were applied. Eligible patients were required to be on stable hormone replacement therapy (HRT) for at least 12 months prior to enrollment. Specifically, male participants were receiving either gonadotropins or testosterone supplementation, while female participants were on a consistent estrogen–progestogen regimen. Biochemical stability on HRT was a prerequisite for inclusion. For male patients, serum total testosterone (tT) levels had to be within the age-specific physiological reference range (300-1000 ng/dL or 10.4-34.7 nmol/L). For female patients on oral or transdermal estroprogestin therapy, stability was defined as serum estradiol (E2) levels within the expected target physiological range (30-150 pg/mL or 110-550 pmol/L). Furthermore, all participants were required to possess full cognitive capacity to comprehend the nature of the study and provide written informed consent.

Due to the rare nature of the pathology, the sample size was not determined a priori. A pragmatic sampling approach was adopted, aiming to enroll ∼10 patients per study group.

A group of healthy volunteers (age > 18 years) was enrolled to serve as a control cohort. Control subjects were included only if they met the aforementioned eugonadal reference ranges and were confirmed to be normosmic following the Sniffin’ Sticks test (22). To facilitate a robust comparative analysis, controls were carefully matched for both age and sex with the KS and nHH groups. All eligible participants who met the inclusion criteria and provided informed consent within the study timeframe were included.

### Study protocol

A comprehensive review of medical records, supplemented by structured patient interviews, was conducted to extract key clinical and demographic data. Specifically, the following information was recorded for participants with CHH: age at diagnosis, age at the commencement of HRT, and the type, duration, and stability of the current therapeutic regimen.

To assess the endocrine profile, serum levels of luteinizing hormone (LH), follicle-stimulating hormone (FSH), tT and free testosterone (fT), E2, prolactin (PRL), and thyroid-stimulating hormone (TSH) were analyzed. For male patients with CHH, blood samples were drawn in the morning (between 8:00 and 10:00 Am) before the daily application for those on transdermal gel, and at trough (immediately prior to the next injection) or mid-interval for those on injectable formulations or gonadotropins. For female patients with CHH, blood samples were collected during the active estrogen phase, immediately before the next scheduled dose or patch replacement. For all controls, fasting blood samples were collected in the morning; specifically, female controls underwent blood sampling during the early follicular phase of their menstrual cycle (typically between days 2 and 5). Hormonal levels were quantified via ADVIA Centaur XP® (Siemens Healthcare Diagnostics Inc., Tarrytown, NY, USA).

Furthermore, information regarding relationship status (eg, single vs in a committed relationship), sexual orientations and potential clinical confounding variables—including comorbidities and medications known to interfere with libido or sexual function—were systematically collected to ensure a robust analysis.

To ensure that differences in psychosexual outcomes were not biased by pre-existing disease severity, retrospective baseline (pretreatment) clinical and biochemical records were extracted. These included history of cryptorchidism and micropenis in males, baseline testicular and ovarian volumes (evaluated by ultrasound and/or orchidometer), baseline gonadotropins, sex steroids, inhibin B, AMH, nonreproductive congenital anomalies, and genetic variant screening [Table S1 (23)].

To explore the multidimensional nature of sexual function, a battery of validated psychometric instruments was administered. These tools were selected to assess distinct, nonoverlapping functional levels:

- Sexual desire inventory-2 (SDI-2): employed to evaluate the motivational system, distinguishing between dyadic (interpersonal) and solitary sexual desire (24);
- Sociosexual orientation inventory-revised (SOI-R): Used to assess the relational dimension, specifically measuring an individual's predisposition toward casual vs committed sexual encounters (25);
- Sexual inhibition/sexual excitation scales (SIS/SES): these scales identify the regulatory “brakes” and “facilitators” of sexual response, serving as a proxy for the mechanisms that translate desire into behavior (26);
- Brief sexual attitudes scale (BSAS): Administered to quantify the cognitive and value-based frameworks of sexuality, helping to determine whether sexual difficulties stem from cultural/cognitive factors rather than biological or regulatory deficits (27).

To ensure a high-resolution evaluation of the psychosexual architecture within the cohort, data analysis was strictly conducted at the subdomain level for each psychometric instrument, avoiding the mathematical aggregation of overarching global scores. The rationale for this approach is rooted in the validated multidimensional nature of the adopted questionnaires. For instance, the SOI-R explicitly dissociates overt sociosexual behavior from cognitive attitudes and sociosexual desire (25). Similarly, the BSAS measures orthogonal constructs which cannot be psychometrically conflated without violating construct validity (27). The SDI-2 independently evaluates dyadic and solitary desire, acknowledging that these drives may operate via different neuro-cognitive pathways (24). Finally, the dual-control model intrinsically requires the separation of excitatory triggers(SES) from distinct inhibitory mechanisms (SIS1 and SIS2) (26). Consequently, subdomain stratification was methodologically essential to accurately distinguish between intact cognitive-attitudinal dimensions and defective physiological or behavioral responses. This subdomain-level approach was also clinically motivated, as it allows clinicians to distinguish between patients whose primary difficulties lie in motivational-cognitive schemas (eg, restrictive sexual attitudes or relational avoidance) and those, such as KS patients, in whom a structurally impaired sensory gateway may selectively disrupt the translation of intact desire into embodied arousal and enacted behavior.

### Statistical analysis

Statistical analyses were performed using SPSS (version 28.0 or later) or R software. Data distribution was assessed for normality using the Shapiro–Wilk test. Descriptive statistics were expressed as mean ± standard deviation (SD) for normally distributed continuous variables, or as median and interquartile range (IQR) for nonparametric data. Categorical variables were presented as frequencies and percentages. To address the primary objective, differences among the 3 study groups (KS, nHH, and healthy controls) were analyzed using one-way ANOVA (with Tukey or Bonferroni posthoc tests) for normally distributed data, or the Kruskal–Wallis test (followed by Dunn test) for nonparametric variables. For 2-group comparative analyses (including clinical subgroups and gender dimorphism evaluations), the independent-samples *t*-test was utilized for normally distributed data, and the Mann–Whitney *U* test was applied for nonparametric datasets.

Categorical clinical data and relationship status were compared using the χ^2^ test or Fisher exact test as appropriate. To isolate the independent contribution of the olfactory deficit from endocrine and demographic factors, multiple linear regression models were employed, adjusting for age, duration of HRT, and relationship status. Statistical significance for all tests was set at a two-tailed *P* < .05.

## Results

### Cohort demographics and hormonal baseline

The study enrolled a total of 38 participants, consisting of 19 patients diagnosed with CHH and 19 healthy control subjects. The overall sample comprised 29 males (76.3%) and 9 females (23.7%). Within the clinical arm, 10 patients presented with the hypo/anosmic phenotype (KS) and 9 were classified as having nHH. All patients with CHH were undergoing stable HRT at the time of the cross-sectional evaluation (Table 1).

**Table 1:** Hormonal profile comparison among patients with KS, patients with nHH, and healthy controls

Variable	KS patients (*n* = 10)	nHH patients (*n* = 9)	Controls (*n* = 19)	*P*-value (KS vs nHH)	*P*-value (patients with CHH vs controls)
Age (years)	40.5 (24.0-43.8)	42.0 (38.0-46.0)	38.0 (32.0-42.0)	.437	.511
Sex (female)	4 (40.0%)	1 (11.1%)	4 (21.1%)	.303	1.000
LH (mIU/mL)	0.20 (0.10-0.35)	0.25 (0.10-0.40)	4.60 (3.20-6.10)	.845	**<.001**
FSH (mIU/mL)	0.35 (0.15-0.50)	0.40 (0.20-0.65)	5.10 (3.80-7.30)	.790	**<.001**
tT (ng/dL) [males]	485.0 (410.0-590.0)	512.0 (435.0-620.0)	545.0 (460.0-675.0)	.612	.425
E2 (pg/mL) [females]	76.5 (55.0-112.0)	81.0 (62.0-118.0)	70.0 (48.0-95.0)	.880	.510
PRL (ng/mL)	8.7 (6.4-11.5)	9.3 (7.1-12.4)	8.9 (6.5-11.8)	.715	.910
TSH (µIU/mL)	1.75 (1.20-2.40)	1.60 (1.10-2.25)	1.70 (1.25-2.35)	.650	.875

Continuous variables are expressed as median (interquartile range, IQR). Categorical variables are expressed as numbers (percentages). Bold values indicate statistical significance.

Abbreviations: CHH, congenital hypogonadotropic hypogonadism; E2, estradiol; FSH, follicle-stimulating hormone; KS, Kallmann syndrome; LH, luteinizing hormone; nHH, normosmic hypogonadotropic hypogonadism; PRL, prolactin; TSH, thyroid-stimulating hormone; tT, total testosterone.

As detailed in Table S1 (23), baseline pretreatment evaluations confirmed that both KS and nHH cohorts exhibited comparable severe hypogonadotropism prior to therapy initiation. Baseline pretreatment testicular volumes in males (median: 2.0 vs 2.0 cm^3^, *P* = .414) and baseline ovarian volumes in females (*P* = .400) did not differ significantly between groups. Furthermore, the prevalence of cryptorchidism (33.3% vs 25.0%, *P* = 1.000), micropenis (16.7% vs 12.5%, *P* = 1.000), and nonreproductive anomalies was evenly distributed across KS and nHH cohorts, ruling out baseline phenotypic disparity as a confounding factor.

Hormonal assessments confirmed the underlying pathophysiology of CHH, demonstrating that gonadotropin levels were profoundly suppressed or blunted in both patient groups compared with healthy controls (LH: *P* < .001; FSH: *P* < .001). Conversely, regular and stable adherence to the therapeutic regimen successfully maintained target physiological eugonadism across all cohorts. No statistically significant differences were observed in serum tT levels among male participants (*P* = .612 for KS vs nHH; *P* = .425 for CHH vs controls) or in E2 levels among female participants during the active therapeutic phase (*P* = .880 for KS vs nHH; *P* = .510 for CHH vs controls). Furthermore, baseline evaluations confirmed euthyroidism and ruled out hyperprolactinemia, as TSH (*P* = .650 for KS vs nHH; *P* = .875 for CHH vs controls) and PRL (*P* = .715 for KS vs nHH; *P* = .910 for CHH vs controls) levels remained well within normal physiological limits without significant intergroup variations.

### Overall comparison between patients with CHH and controls

The manifestation of CHH under stable HRT did not induce an overall dampening of psychosexual response patterns when compared with healthy individuals (Table 2; Fig. 1). Regarding the dual-control regulatory mechanisms, no macrostructural deviations were detected between the cohorts. Specifically, the aggregated SES scores (2.73 ± 0.62 in CHH vs 2.89 ± 0.37 in controls, *P* = .299) and sexual inhibition scores showed no statistically significant deviations between the 2 main arms, both for inhibition from distraction (SIS1: 2.76 ± 0.50 vs 2.65 ± 0.51, *P* = .354) and inhibition from threat or anxiety (SIS2: 2.71 ± 0.62 vs 2.59 ± 0.52, *P* = .412). Furthermore, patients shared comparable attitudinal frameworks across all subdomains of the BSAS, including permissiveness (*P* = .759), birth control (*P* = .145), communion (*P* = .455), and instrumentality (*P* = 1.000). Baseline sexual drive evaluated via the SDI-2 was also broadly preserved, showing no significant differences for either dyadic (4.28 ± 2.23 vs 4.15 ± 2.31, *P* = .930) or solitary desire (4.38 ± 2.69 vs 4.00 ± 2.53, *P* = .737). Similarly, psychometric scores derived from the SOI-R revealed stable baseline profiles across the behavior (*P* = .403), attitude (*P* = .111), and desire (*P* = .647) subscales. The sole statistically significant variance identified in this global analysis was the total number of different sexual partners over the preceding 12 months, which was significantly lower in the CHH cohort than in the healthy control group (1.84 vs 2.58, *P* = .019).

**Figure 1:**
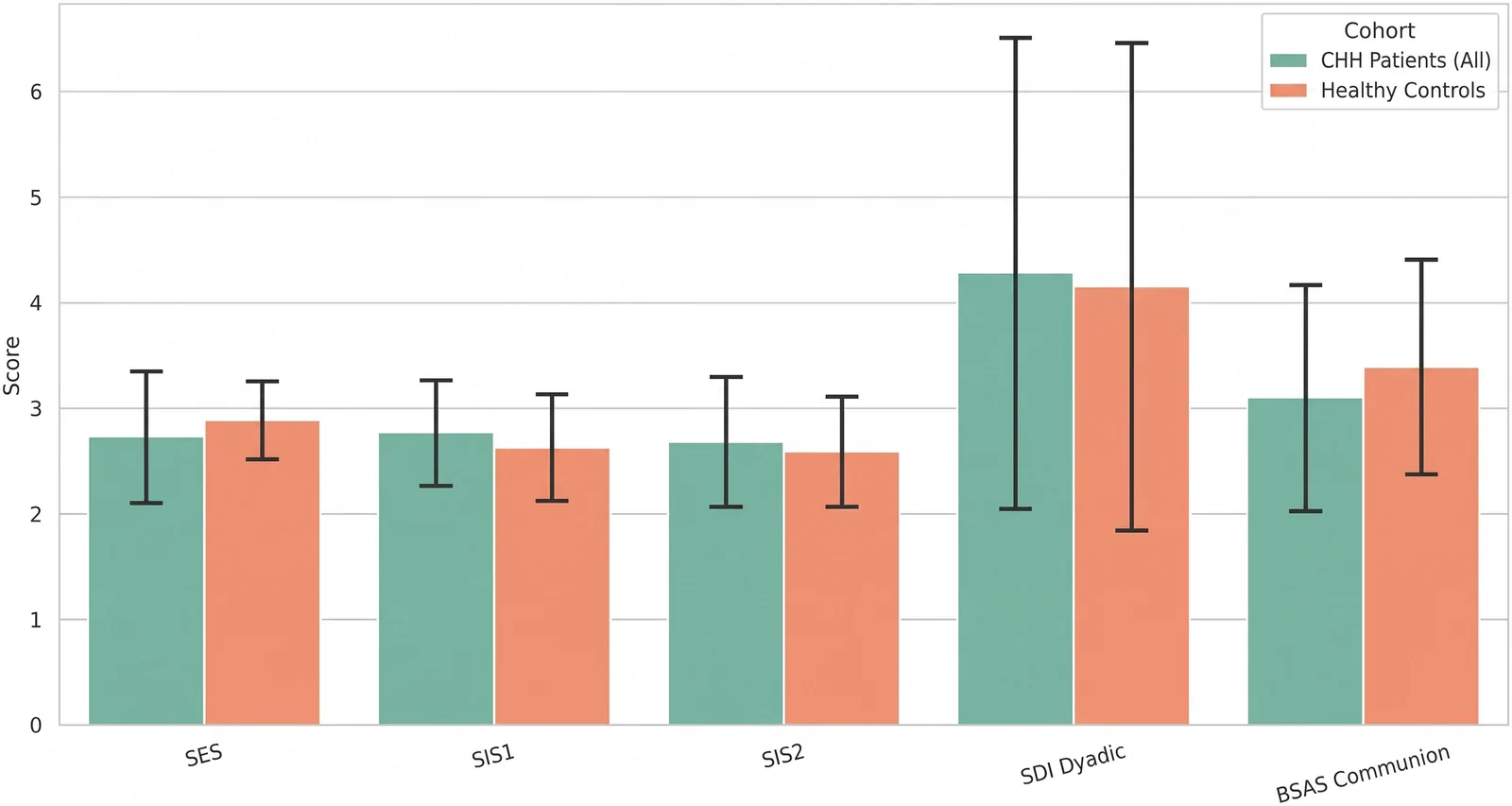
Global psychosexual comparison between treated CHH patients and healthy controls. Bar plots represent the mean scores across key psychometric subdomains: sexual excitation (SES), sexual inhibition (SIS1 and SIS2), dyadic desire (SDI dyadic), and communion (BSAS communion). Error bars indicate standard deviations. No statistically significant differences were observed between the clinical cohort (*n* = 19) and healthy controls (*n* = 19) across these parameters (*P* > .05, Mann–Whitney *U* test), suggesting that adequate hormone replacement therapy effectively restored baseline psychosexual frameworks.

**Table 2:** Global comparison between patients with CHH and healthy controls

Variable	CHH (*n* = 19)	Controls (*n* = 19)	*P*-value
SES	2.73 ± 0.62	2.89 ± 0.37	.299
SIS1	2.76 ± 0.50	2.65 ± 0.51	.354
SIS2	2.71 ± 0.62	2.59 ± 0.52	.412
BSAS: permissiveness	2.69 ± 0.99	2.93 ± 1.00	.759
BSAS: birth control	3.49 ± 1.23	3.00 ± 0.96	.145
BSAS: communion	3.10 ± 1.07	3.39 ± 1.02	.455
BSAS: instrumentality	2.73 ± 0.71	2.77 ± 0.80	1.000
SOI-R: behavior	2.79 ± 4.47	4.76 ± 7.29	.403
SOI-R: attitude	5.00 ± 2.38	6.12 ± 1.90	.111
SOI-R: desire	3.04 ± 1.97	3.56 ± 2.62	.647
SDI-2: dyadic desire	4.28 ± 2.23	4.15 ± 2.31	.930
SDI-2: solitary desire	4.38 ± 2.69	4.00 ± 2.53	.737
Number of different sexual partners (last 12 months)	1.84	2.58	.**019**

Dichotomous variables are expressed as numbers (percentages); continuous variables are expressed as means ± standard differences. Bold values indicate statistical significance.

Abbreviations: BSAS, brief sexual attitudes scale; CHH, congenital hypogonadotropic hypogonadism; SES, sexual excitation scale; SIS, sexual inhibition scale; SOI-R, sociosexual orientation inventory-revised.

### Comparison between KS, nHH, and controls

#### Patients with hypoanosmia (KS) vs patients with normosmia (nHH and healthy controls)

To isolate the specific independent contribution of the olfactory deficit from general endocrine and demographic factors, stratified analyses were performed by comparing hypoanosmic patients against normosmic cohorts (Table 3). When comparing hypoanosmic patients (KS) against the total normosmic sample, which pooled both nHH patients and healthy controls, a drastic reduction in overall sexual arousability was recorded. This was indicated by significantly lower scores on the SES subscale among anosmic individuals (2.34 ± 0.46 vs 2.97 ± 0.42, *P* = .001). This sensory-specific excitatory deficit directly corresponded to altered sociological behavior, as evidenced by a marked collapse in objective sociosexual behavior scores (SOI-R Behavior: 0.70 ± 0.66 in KS vs 4.88 ± 6.73 in normosmics, *P* = .015). Conversely, no significant variations emerged between KS and total normosmics regarding regulatory inhibition mechanisms (SIS1: *P* = .702; SIS2: *P* = .654), cognitive sexual attitudes across all BSAS subscales, or subjective sexual desire metrics (SDI-2 dyadic desire: *P* = .416; SDI-2 solitary desire: *P* = .465).

**Table 3:** Comparison between patients with hypoanosmia (KS) and normosmia (nHH + controls)

Psychometric subscale	Normosmic individuals (*n* = 28)	Hypoanosmic patients (*n* = 10)	*P*-value
SES	2.97 ± 0.42	2.34 ± 0.46	.**001**
SIS1	2.69 ± 0.56	2.73 ± 0.32	.702
SIS2	2.62 ± 0.63	2.70 ± 0.33	.654
BSAS: permissiveness	2.90 ± 1.02	2.56 ± 0.89	.573
BSAS: birth control	3.21 ± 1.11	3.33 ± 1.19	.726
BSAS: communion	3.36 ± 1.10	2.90 ± 0.82	.219
BSAS: instrumentality	2.70 ± 0.82	2.88 ± 0.51	.414
SOI-R: behavior	4.88 ± 6.73	0.70 ± 0.66	.**015**
SOI-R: attitude	5.94 ± 2.12	4.50 ± 2.16	.075
SOI-R: desire	3.58 ± 2.44	2.50 ± 1.71	.261
SDI-2: dyadic desire	4.35 ± 2.28	3.84 ± 2.21	.416
SDI-2: solitary desire	4.37 ± 2.55	3.70 ± 2.77	.465

Variables are expressed as means ± standard differences. Bold values indicate statistical significance.

Abbreviations: BSAS, brief sexual attitudes scale; CHH, congenital hypogonadotropic hypogonadism; SES, sexual excitation scale; SIS, sexual inhibition scale; SOI-R, sociosexual orientation inventory-revised.

#### Patients with KS vs patients with nHH

By restricting the comparative analysis exclusively to the clinical arm of patients with CHH, a profound phenotypical divergence was isolated between the KS and nHH subgroups within domains linked to limbic arousal processing (Table 4; Fig. 2). KS patients exhibited a highly significant impairment in the sexual excitation system compared with nHH patients, with severely reduced SES scores (2.34 ± 0.46 vs 3.16 ± 0.48, *P* = .002). This selective excitatory failure strongly correlated with a reduction in active sexual initiation, resulting in a dramatic drop in overt sociosexual behaviors (SOI-R behavior: 0.70 ± 0.66 vs 5.11 ± 5.75, *P* = .021). Strikingly, their cognitive-theoretical desire measured by the SDI-2 and value-based attitudes assessed via the BSAS remained perfectly preserved and statistically homogeneous, showing no significant differences in dyadic desire (*P* = .288), solitary desire (*P* = .235), sociosexual attitudes (*P* = .346), sociosexual desire (*P* = .280), or any attitudinal subscales. These findings highlight a severe, sensory-specific dissociation between an intact psychosexual intent and a defective physiological arousability within the KS phenotype.

**Figure 2:**
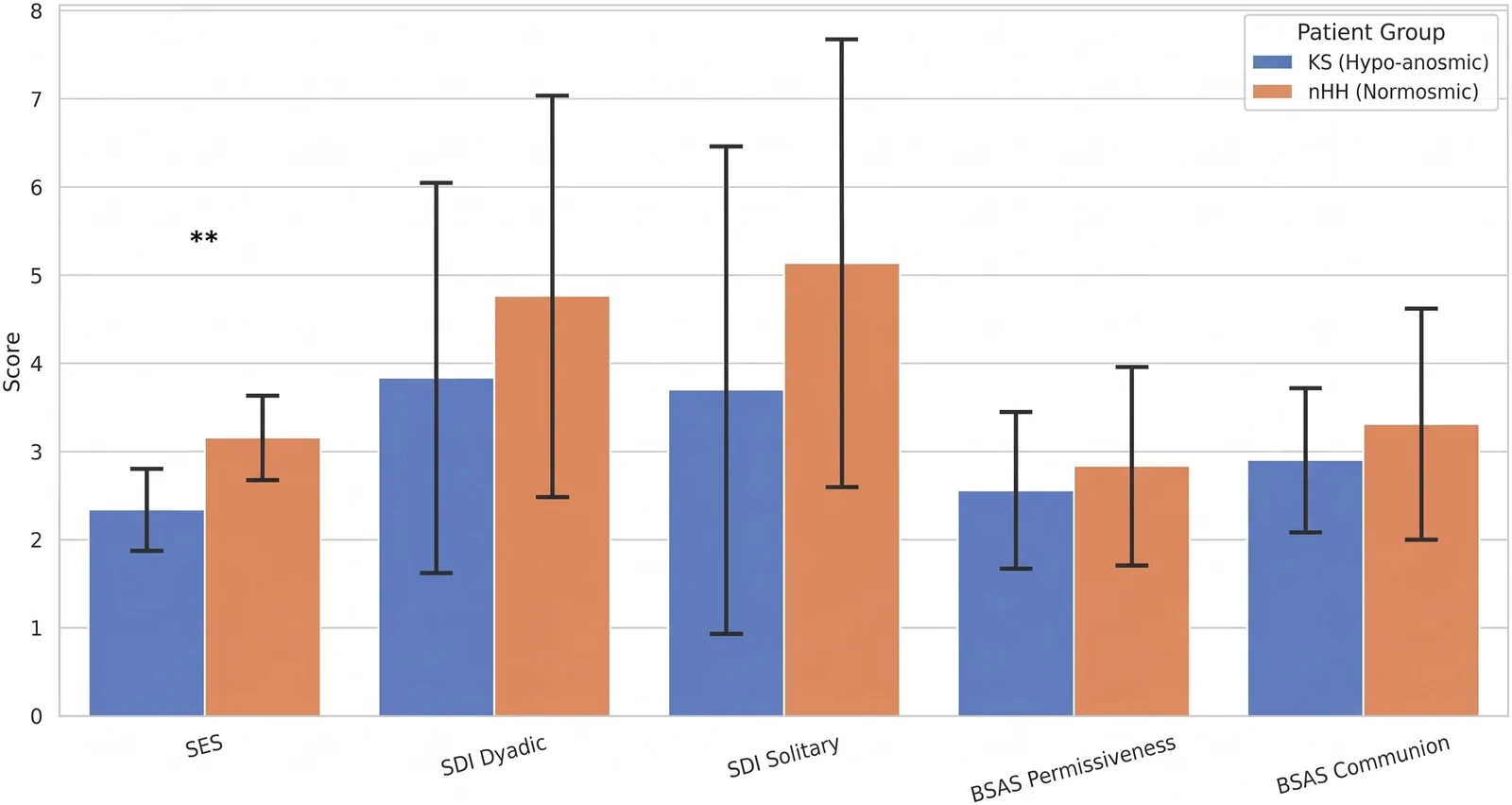
Dissociation of sexual arousability and cognitive desire in KS. Mean psychometric scores comparing hypoanosmic patients (KS, *n* = 10) with normosmic patients (nHH, *n* = 9). While cognitive sexual desire (SDI dyadic and solitary) and sexual attitudes (BSAS permissiveness and communion) show no significant differences between the 2 phenotypes, KS patients exhibit a highly significant impairment in the sexual excitation system (SES, *P* = .002). Error bars represent standard deviations. ** *P* < .01.

**Table 4:** Comparison between patients with KS and nHH

Psychometric subscale	nHH patients (*n* = 9)	KS patients (*n* = 10)	*P*-value
SES	3.16 ± 0.48	2.34 ± 0.46	.**002**
SIS1	2.81 ± 0.67	2.73 ± 0.32	.870
SIS2	2.67 ± 0.86	2.70 ± 0.33	.594
BSAS: permissiveness	2.83 ± 1.13	2.56 ± 0.89	.540
BSAS: birth control	3.67 ± 1.33	3.33 ± 1.19	.510
BSAS: communion	3.31 ± 1.31	2.90 ± 0.82	.367
BSAS: instrumentality	2.56 ± 0.89	2.88 ± 0.51	.317
SOI-R: behavior	5.11 ± 5.75	0.70 ± 0.66	.**021**
SOI-R: attitude	5.56 ± 2.61	4.50 ± 2.16	.346
SOI-R: desire	3.63 ± 2.16	2.50 ± 1.71	.280
SDI-2: dyadic desire	4.76 ± 2.28	3.84 ± 2.21	.288
SDI-2: solitary desire	5.14 ± 2.54	3.70 ± 2.77	.235

Variables are expressed as means ± standard differences. Bold values indicate statistical significance.

Abbreviations: BSAS, brief sexual attitudes scale; CHH, congenital hypogonadotropic hypogonadism; KS, Kallmann syndrome; nHH, normosmic hypogonadotropic hypogonadism; SES, sexual excitation scale; SIS, sexual inhibition scale; SOI-R, sociosexual orientation inventory-revised.

### Overall comparison between male and female participants

Dimorphic differences in human sexuality were initially assessed across the total global sample of 38 participants, validating the psychometric reliability of the utilized instruments by replicating well-established gender variations (Table 5; Fig. 3). Male participants reported significantly higher scores in permissive sexual attitudes (BSAS permissiveness: 3.05 ± 0.93 vs 2.03 ± 0.77, *P* = .007) and viewed sexuality through a significantly more instrumental framework (BSAS instrumentality: 2.90 ± 0.69 vs 2.24 ± 0.73, *P* = .047) than female participants. Furthermore, males demonstrated substantially higher levels of sociosexual desire for uncommitted intimacy (SOI-R Desire: 3.77 ± 2.38 vs 1.78 ± 1.14, *P* = .016), alongside a strong trend toward higher solitary sexual desire (SDI-2 solitary desire: 4.69 ± 2.31 vs 2.58 ± 2.91, *P* = .052).

**Figure 3:**
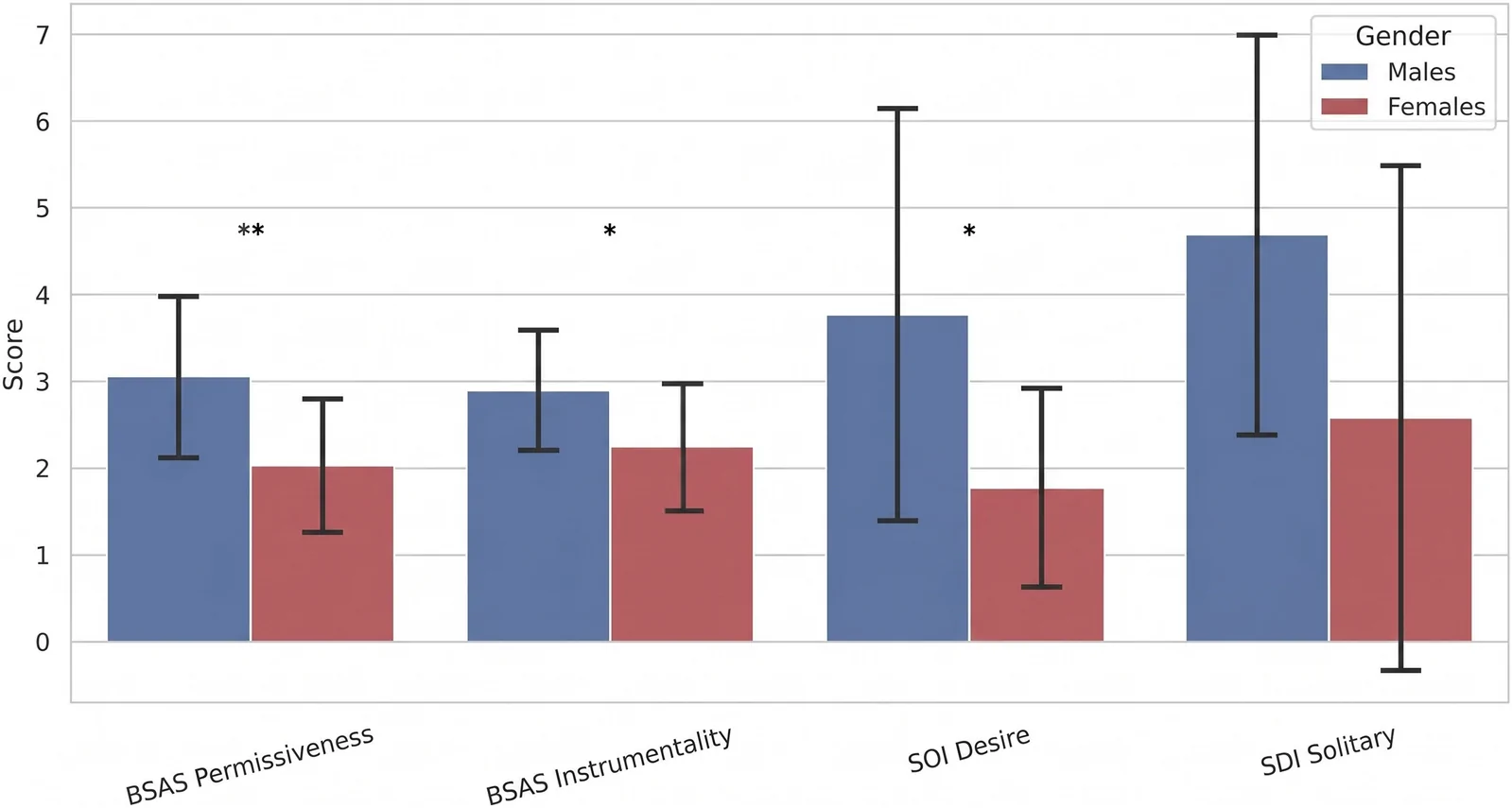
Preservation of evolutionary gender dimorphism across the total sample. Mean scores comparing male (*n* = 29) and female (*n* = 9) participants across the pooled cohort (patients with CHH and controls). Males scored significantly higher in domains reflecting sociosexual permissiveness, instrumentality, and solitary desire, validating the psychometric reliability of the instruments utilized. Error bars denote standard deviations. * *P* < .05; ** *P* < .01.

**Table 5:** Sexual dimorphism (male vs female) in the global sample (patients with CHH and controls)

Psychometric subscale	Males (*n* = 29)	Females (*n* = 9)	*P*-value
SES	2.88 ± 0.40	2.57 ± 0.75	.110
SIS1	2.61 ± 0.49	2.98 ± 0.45	.063
SIS2	2.54 ± 0.55	2.94 ± 0.52	.079
BSAS: permissiveness	3.05 ± 0.93	2.03 ± 0.77	.**007**
BSAS: birth control	3.40 ± 0.98	2.74 ± 1.43	.246
BSAS: communion	3.35 ± 1.00	2.91 ± 1.16	.318
BSAS: instrumentality	2.90 ± 0.69	2.24 ± 0.73	.**047**
SOI-R: behavior	4.35 ± 6.67	1.93 ± 2.91	.255
SOI-R: attitude	5.69 ± 2.20	5.15 ± 2.27	.371
SOI-R: desire	3.77 ± 2.38	1.78 ± 1.14	.**016**
SDI-2: dyadic desire	4.61 ± 2.08	2.94 ± 2.38	.068
SDI-2: solitary desire	4.69 ± 2.31	2.58 ± 2.91	.052

Variables are expressed as means ± standard differences. Bold values indicate statistical significance.

Abbreviations: BSAS, brief sexual attitudes scale; CHH, congenital hypogonadotropic hypogonadism; SES, sexual excitation scale; SIS, sexual inhibition scale; SOI-R, sociosexual orientation inventory-revised.

Importantly, these dimorphic traits remained robustly unaltered even when analyzing the hypogonadal clinical cohort under stable HRT in isolation (Table 6). Within the CHH patient group, male patients retained significantly higher levels of sexual permissiveness compared with female patients (BSAS permissiveness: 3.03 ± 0.81 vs 1.74 ± 0.86, *P* = .018) and displayed significantly higher overt sociosexual behaviors (SOI-R behavior: 3.62 ± 4.98 vs 0.47 ± 0.56, *P* = .050). Borderline differences tracking with standard sexual dimorphism were also observed for sociosexual desire (SOI-R desire: 3.52 ± 1.93 vs 1.67 ± 1.49, *P* = .053) and solitary desire (SDI-2 solitary desire: 5.20 ± 1.95 vs 2.10 ± 3.38, *P* = .086). Conversely, regulatory response mechanisms (SES, SIS1, and SIS2) and cognitive relationship values like sexual communion demonstrated complete gender homogeneity within the clinical population, showing no statistically significant variations between male and female patients.

**Table 6:** Sexual dimorphism (male vs female) in the group of patients with CHH

Psychometric subscale	Males (*n* = 14)	Females (*n* = 5)	*P*-value
SES	2.83 ± 0.43	2.45 ± 1.00	.179
SIS1	2.66 ± 0.44	3.08 ± 0.59	.114
SIS2	2.55 ± 0.56	3.05 ± 0.67	.149
BSAS: permissiveness	3.03 ± 0.81	1.74 ± 0.86	.**018**
BSAS: birth control	3.76 ± 0.94	2.73 ± 1.74	.243
BSAS: communion	3.26 ± 1.09	2.64 ± 0.96	.285
BSAS: instrumentality	2.84 ± 0.66	2.40 ± 0.83	.422
SOI-R: behavior	3.62 ± 4.98	0.47 ± 0.56	.050
SOI-R: attitude	5.31 ± 2.43	4.13 ± 2.22	.178
SOI-R: desire	3.52 ± 1.93	1.67 ± 1.49	.053
SDI-2: dyadic desire	4.89 ± 1.59	2.55 ± 3.01	.151
SDI-2: solitary desire	5.20 ± 1.95	2.10 ± 3.38	.086

Variables are expressed as means ± standard differences. Bold values indicate statistical significance.

Abbreviations: BSAS, brief sexual attitudes scale; CHH, congenital hypogonadotropic hypogonadism; SES, sexual excitation scale; SIS, sexual inhibition scale; SOI-R, sociosexual orientation inventory-revised.

### Multiple linear regression analysis for the global sample

To evaluate the independent impact of olfactory function on sexual excitation across the entire study population (*n* = 38), a multiple linear regression model was constructed (Table 7). The SES score was entered as the dependent variable. The primary predictor was olfactory status, entered as a dummy variable (0 = normosmic; 1 = hypoanosmic/KS). To control for potential confounding demographic factors, biological sex (0 = female and 1 = male) and chronological age (in years) were entered simultaneously as covariates. The overall model was statistically significant, explaining a notable proportion of the variance in sexual excitation scores (*R*^2^ = 0.339, Adjusted *R*^2^ = 0.281, *F*(3, 34) = 5.820, and *P* = .003). After adjusting for age and sex, olfactory status emerged as a significant independent predictor of sexual excitation (*β* = −.499, *t* = −3.413, and *P* = .002). Conversely, neither chronological age (*β* = .113, *t* = 0.768, and *P* = .448) nor biological sex (*β* = .181, *t* = 0.768, and *P* = .448) reached statistical significance within this multipredictor framework.

**Table 7:** Multiple linear regression model predicting SES scores in the global sample

Predictor variable	Unstandardized *B*	Standard error	Standardized *β*	*t*-value	*P*-value	95% CI lower	95% CI upper
Constant	2.551	0.399	—	6.386	<.001	1.739	3.362
Olfactory status (hypoanosmic)	−0.570	0.167	−.499	−3.413	.002	−0.909	−0.230
Sex (male)	0.214	0.177	.181	1.204	.237	−0.147	0.574
Age (years)	0.006	0.008	.113	0.768	.448	−0.011	0.023

## Discussion

The primary objective of this study was to distinguish the impact of the endocrine dysfunction inherent to CHH from the specific contribution of the olfactory deficit present in KS on sexual desire and relationships. Our findings indicate that adequate and stable HRT effectively restores the overarching psychosexual baseline; overall, the treated CHH cohort did not exhibit a dampening of psychosexual response patterns when compared with healthy controls, except for the reduced number of sexual partners. Psychologically, the subtle constraints in partner turnover may still reflect long-term illness narratives, cautious partner selection, or residual health-related insecurity acquired during delayed puberty in patients with CHH (28). A profound phenotypical divergence emerged when isolating the olfactory variable by comparing hypo/anosmic KS patients directly with nHH patients. KS patients exhibited a highly significant impairment in their sexual excitation system, which strongly correlated with a dramatic drop in overt sociosexual behaviors. Strikingly, despite these behavioral and arousal deficits, the cognitive-theoretical desire and value-based sexual attitudes of KS patients remained perfectly preserved and statistically homogeneous with their normosmic counterparts. These results highlight a severe, sensory-specific dissociation within the KS phenotype: an intact psychosexual intent coupled with a defective physiological arousability. Multiple linear regression analysis further reinforced this, demonstrating that olfactory status serves as a significant independent predictor of sexual excitation, overriding potential demographic confounders such as chronological age and biological sex.

From a psychological standpoint, the collapse of sociosexual behavior in KS, despite preserved desire and attitudes, suggests a chronic “intention–action gap” that is not explained by inhibitory tendencies but by a lack of sensory reinforcement and bodily confidence. Qualitative work on anosmia indicates that individuals often experience heightened concern about their own body odor, reduced perceived attractiveness, and a sense of disconnection from their partner's unique scent, all factors that can undermine sexual initiative and reinforce withdrawal from casual or exploratory encounters (12, 14, 15).

Clinically, this phenotype is compatible with a form of “sensory-specific learned helplessness” in the erotic domain: over time, repeated failures to experience reliable bodily arousal in response to sexual cues may condition KS patients to down-regulate behavioral initiative, while their cognitive and value-based erotic schemas remain intact and, in some cases, even idealized. Such a dissociation is likely to expose individuals to cumulative frustration, doubts about their own sexual adequacy, and relational misunderstandings, especially when partners interpret reduced initiation as disinterest rather than a structurally constrained arousal pathway (6, 14, 16).

The most striking finding of this investigation is the clear, sensory-specific dissociation between an intact psychosexual intent and a defective physiological arousability within the KS phenotype. While cognitive-theoretical sexual desire (measured via the SDI-2) and value-based attitudes (assessed via the BSAS) remained perfectly preserved and statistically homogeneous between the KS and nHH cohorts, the SES score collapsed in the anosmic group. This phenomenon can be elucidated through a neurobiological lens. Exogenous sex steroids successfully activate the hormone-dependent hypothalamic pathways that drive cognitive libido, sociosexual desire, and value frameworks (29). However, they cannot restore or bypass the permanently severed connections within the olfactory-limbic-hypothalamic network resulting from the embryonic failure of GnRH and olfactory neuron migration. Olfaction acts as an essential sensory gateway for sexual arousability in humans (6). Without this structural gateway, KS patients face an immediate block in translating their intact cognitive desire into physiological excitation (SES) and subsequent proactive sociosexual behavioral initiation (SOI-R Behavior). This independent, overriding effect of the sensory deficit was robustly confirmed by our multiple linear regression model, where olfactory status emerged as a powerful independent predictor of sexual excitation (*β* = −.499; *P* = .002), completely eclipsing chronological age and biological sex.

A major strength of this study lies in its strict methodological design, which successfully isolated the olfactory variable by utilizing a highly specific comparative framework of KS, nHH, and healthy controls. The application of stringent inclusion criteria—requiring all clinical participants to be on stable HRT for at least 12 months with biochemically confirmed eugonadism—minimized confounding variables related to hormonal fluctuations. Furthermore, the strategic decision to strictly analyze the battery of validated psychometric instruments at the subdomain level, rather than relying on aggregated global scores, allowed for a high-resolution evaluation capable of detecting the dissociation between cognitive intent and physiological arousal.

The primary limitation of this study is its small sample size, comprising 19 patients with CHH. Because of the rare nature of the pathology, the sample size was not determined a priori, which may limit the generalizability of the findings. Furthermore, while our multiple linear regression model identified olfactory status as a robust independent predictor of sexual excitation, entering 3 multipredictor covariates (olfactory status, biological sex, and age) into a model with 38 subjects introduces a potential risk of statistical overfitting. Consequently, these regression metrics should be viewed as exploratory, serving as a methodological framework for future, larger-scale validation studies. A critical methodological constraint pertains to the sex distribution of our clinical cohort (14 males and 5 females, including 4 KS females and 1 nHH female). This distribution strictly mirrors the well-established epidemiological asymmetry of CHH and KS, which has a recognized male predominance (∼4-5:1). Due to the very small female subsample, conducting separate inferential subgroup analyses (eg, KS vs nHH separately for each sex) would be statistically underpowered and methodologically unfeasible. To rigorously address, this limitation without compromising statistical validity, we adopted a 2-fold approach: first, we evaluated overall sexual dimorphism across cohorts (Tables 5 and 6); second, we included biological sex as an explicit covariate in our multiple linear regression model (Table 7). In this model, olfactory status remained a robust, independent predictor of sexual excitation (*P* = .002), whereas sex was not significant (*P* = .237). Nonetheless, we explicitly acknowledge that our findings should be considered preliminary trends rather than definitive evidence of gender homogeneity or divergence in regulatory psychosexual mechanisms. Additionally, the cross-sectional observational design of this study captures only a single chronological snapshot of the patients’ psychosexual profiles, precluding the determination of absolute causal trajectories over time. Lastly, because the entire clinical cohort was recruited from a single specialized rare disease outpatient clinic in Catanzaro, we cannot completely rule out localized sociocultural or geographic confounders. Sexual attitudes, values, and relationship dynamics—as evaluated via the BSAS and SOI-R frameworks—are highly sensitive to cultural environments, meaning our single-center findings require multicenter replication across socioculturally diverse populations.

From a clinical standpoint, these data demand a paradigm shift in how endocrinologists define therapeutic success in CHH and KS. Traditionally, clinical management focuses almost exclusively on reaching target physiological levels of serum tT or E2, alongside inducing physical secondary sexual characteristics and fertility. While achieving eugonadism successfully resolves systemic metabolic and basic reproductive needs, our findings underscore that endocrinological normalization is an incomplete proxy for holistic patient well-being. The persistent gap between intact cognitive intent and impaired physical arousal leaves KS patients highly vulnerable to silent distress and interpersonal frustration.

Consequently, clinicians should move beyond the biochemical checklist and systematically include brief, validated screening for sexual distress, depressive symptoms, and relational dissatisfaction in the routine follow-up of KS. When clinically indicated, referral to sex therapy or couple-based interventions can help patients and partners reframe arousal difficulties as a predictable consequence of a structural sensory deficit rather than as individual failure, and coconstruct erotic scripts that rely more on visual, tactile, and cognitive stimulation. Providing targeted psychoeducation during the transition from pediatric to adult endocrinology is vital to normalize the possibility of an intention-arousal gap and to equip young adults with concrete communicative strategies for disclosing these difficulties to current or future partners.

Looking forward, multicenter, longitudinal trials are necessary to track the trajectory of psychosexual dimensions from the initiation of HRT through long-term maintenance in both KS and nHH cohorts. Expanding the cohort to include culturally diverse backgrounds will further establish the global validity of the cognitive resilience observed here. Finally, future translational and interventional research should explore neurosensory compensatory mechanisms. Specifically, investigators should examine whether reinforcing other sensory modalities—such as visual, cognitive, or tactile stimuli—or utilizing targeted psychotherapeutic approaches can effectively bypass the structurally compromised olfactory-limbic pathways to enhance physiological arousability and improve the quality of life for individuals living with KS.

## Conclusions

This cross-sectional study suggests that while stable, long-term HRT effectively rescues the global psychosexual architecture in patients with CHH, it remains insufficient to fully restore the complete sexual experience in the presence of a lifelong olfactory defect. Isolating the olfactory variable reveals a profound, sensory-specific dissociation unique to the KS phenotype: cognitive-theoretical sexual desire and value-based attitudes are perfectly preserved, yet the physiological sexual excitation system is severely compromised, directly corresponding to a marked collapse in overt sociosexual behaviors. Olfactory status seems to serve as a powerful independent predictor of sexual excitation, overriding standard demographic and endocrine factors.

Ultimately, these findings underscore that achieving target endocrine ranges may be an incomplete proxy for holistic therapeutic success in KS. Clinicians should expand care to routinely integrate psychosexual counseling and tailored psychoeducation into standard management, thereby mitigating the silent distress and frustration stemming from the persistent gap between intact cognitive intent and impaired physical arousal. Psychologically, KS should therefore be understood not merely as a variant of hypogonadism but as a lifelong condition in which a structural sensory deficit shapes sexual self-experience, intimate relationships, and mental health, calling for integrated endocrine, psychological, and sexological care pathways.

## Data Availability

Some or all datasets generated during and/or analyzed during the current study are not publicly available but are available from the corresponding author on reasonable request.
